# Clinical and Molecular Characterization of NF1 Patients: Single-Center Experience of 32 Patients From China

**DOI:** 10.1097/MD.0000000000003043

**Published:** 2016-03-11

**Authors:** Lude Zhu, Yunfeng Zhang, Hanxing Tong, Minhua Shao, Yong Gu, Xufeng Du, Peiru Wang, Lei Shi, Linglin Zhang, Mingye Bi, Xiuli Wang, Guolong Zhang

**Affiliations:** From the Institute of Photomedicine, Shanghai Skin Disease Hospital, Tongji University School of Medicine (LZ, YZ, PW, LS, LZ, XW, GZ); Department of General Surgery, Zhong Shan Hospital, Fu Dan University (HT), Shanghai; and Department of Dermatology, Nanjing Medical University, Affiliated Wuxi People's Hospital, Wuxi, Jiangsu (MS, YG, XD, MB), China.

## Abstract

Neurofibromatosis type 1 (NF1) is a hereditary disorder caused by mutations in the *NF1* gene. Detecting mutation in *NF1* is hindered by the gene's large size, the lack of mutation hotspots, and the presence of pseudogenes.

Our goal was to establish a sensitive, feasible, and comparatively economical protocol to detect *NF1* mutations using blood samples.

We developed a method to screen patients for mutations. Thirty-two NF1 patients from 32 unrelated families and 120 unrelated population-match controls were investigated in this study. Specific primers were designed for *NF1* to avoid pseudogenes. *NF1* mutations were detected by sequencing at the deoxyribonucleic acid (DNA) and complementary DNA (cDNA) levels, and multiplex ligation-dependent probe amplification (MLPA) and familial segregation analyses were used.

Forty-four specific primers designed according to the *NF1* structure were successfully used for polymerase chain reaction (PCR) and DNA sequencing, which was more feasible and useful than cDNA sequencing. Thirty distinct *NF1* mutations were identified in 32 patients. Thirteen mutations were novel and most were frameshift mutations (33.3%). Mutations were detected at a rate of 93.8%.

Our study suggests that this sensitive, feasible, and comparatively economical protocol is effective for the detection of *NF1* mutations.

## INTRODUCTION

Neurofibromatosis type 1 (NF1; OMIM 162200) is one of the most common genetic disorders (incidence 1:2500–3000)^1^ and is predominantly characterized by multiple café au-lait spots (CALs) and skin neurofibromas, which are attributed to defects in the tumor suppressor gene *NF1*. The *NF1* gene (17.q11.2, 280-kb genomic deoxyribonucleic acid [DNA]) consists of 57 constitutive and at least 3 alternatively spliced exons (9br, 23a, and 48a).^2–4^ The locus has one of the highest spontaneous mutation rates^5^ and nearly half of all NF1 cases are caused by de novo mutations.^6^ More than 1000 distinct *NF1* mutations have been reported; these are summarized in the Human Gene Mutation Database (HGMD).^7^ However, it is difficult to detect mutations in *NF1* owing to the large size of the gene, the presence of pseudogenes, and the lack of mutation hotspots, despite the development of several protocols.^8^

Severe phenotypes have been described in patients with large deletions (∼5–10%) in the *NF1* region, including learning disabilities, facial dysmorphic features, and cardiovascular malformations.^9–11^ For patients with intragenic *NF1* mutations (representing more than 90% of cases),^12–15^ no clear allele-phenotype correlations have been established to date. Accordingly, a molecular analysis of *NF1* is necessary to improve our understanding of the genetic basis of NF1.

The aim of the present study was to establish a sensitive, feasible, and comparatively economical protocol to detect *NF1* mutations using blood samples. Mutation and phenotype analyses were performed in 32 patients to gain further insight into NF1 genotype–phenotype correlations and to contribute additional data to HGMD.

## METHODS

### Patients

Thirty-two NF1 patients from 32 unrelated families were investigated in this study. Nine patients (28.1%) were familial cases and the others were sporadic cases (71.9%). A formal diagnosis of NF1 was made when an individual had 2 or more of the following features in the absence of another diagnosis: 6 or more CALs, axillary or inguinal freckling, 2 or more Lisch nodules, optic glioma, 2 or more neurofibromas of any type or 1 plexiform neurofibroma, a first-degree relative with NF1, or distinctive skeletal abnormalities, such as scoliosis.^16^ The study protocol was approved by the Shanghai Skin Disease Hospital. Written informed consent was obtained from all patients.

### DNA and RNA Extraction

Genomic DNA was extracted from peripheral blood and used as a template for polymerase chain reaction (PCR) amplification of all 58 exons of the *NF1* gene and flanking regions. Total RNA extraction from peripheral blood lymphocytes and reverse transcription were performed according to the manufacturer's instructions (Invitrogen, Carlsbad, CA and MBI Fermentas, Vilnius, Lithuania).

To prevent illegitimate splicing, blood samples were processed after venipuncture with a maximum delay of 4 h and samples were not stored at 4°C.^17,18^ Reverse transcription was performed using 500 ng of total RNA isolated and random hexamers with a First-Strand complementary DNA (cDNA) Synthesis Kit for RT-PCR (AMV) (Roche Applied Science, Indianapolis, IN). The entire coding region of the *NF1* gene was amplified in 23 overlapping fragments by PCR in a 25-μL final reaction mix containing 1.5 μL of cDNA as the template, 5 pmol each primer, 200 μmol/L dNTPs, and 1× reaction buffer with 1.5 mmol/L MgSO_4_ and 1.25 U Optimase Polymerase (Transgenomic, Crewe, UK and Santa Clara, CA). Oligo 6.1 software was used to design primers.

### Primer Design for DNA Sequencing

Using human genome data, Yu et al^19^ obtained the full sequence of all 7 *NF1* pseudogenes, which are partial duplications of the functional *NF1* gene and bear large internal deletions. Based on differences between the sequences of the *NF1* gene and its pseudogenes, specific primers were designed (Table 1  ) to avoid pseudogenes. Some of the PCR products were too long for DNA sequencing, and sites were selected randomly to design additional specific primers to sequence long PCR products. Moreover, nested PCR protocols were adopted to amplify authentic exon 36 in the *NF1* gene. In summary, all coding sequences and the exon–intron boundary sequences of *NF1* were amplified successfully and specifically.

**TABLE 1:**
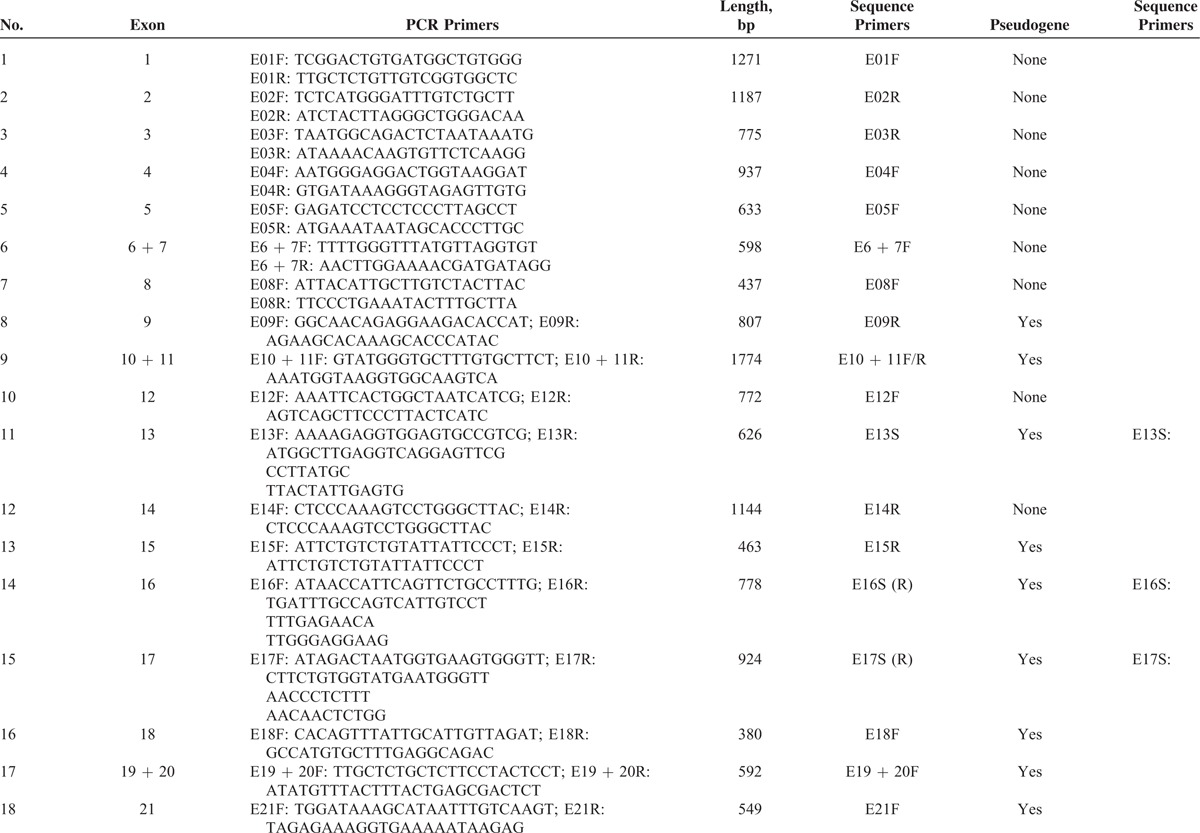
DNA Primers

**TABLE 1 (Continued):**
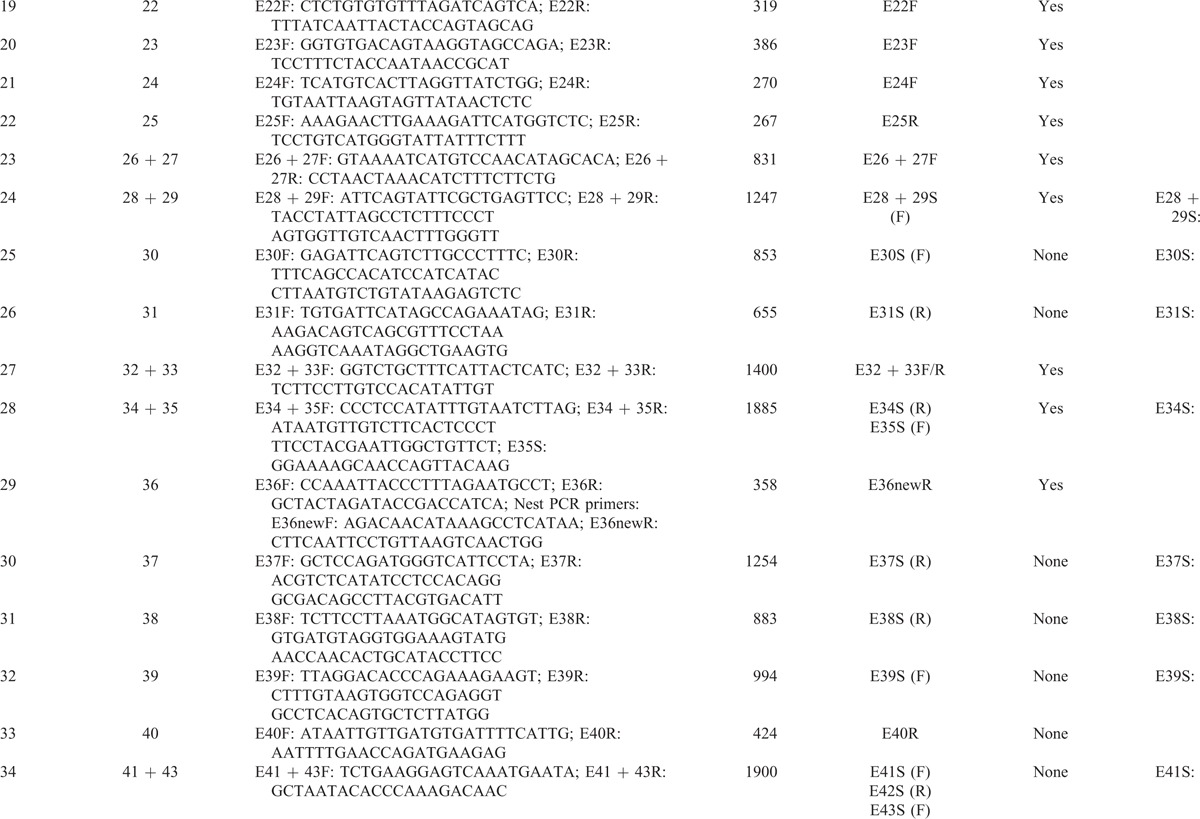
DNA Primers

**TABLE 1 (Continued):**
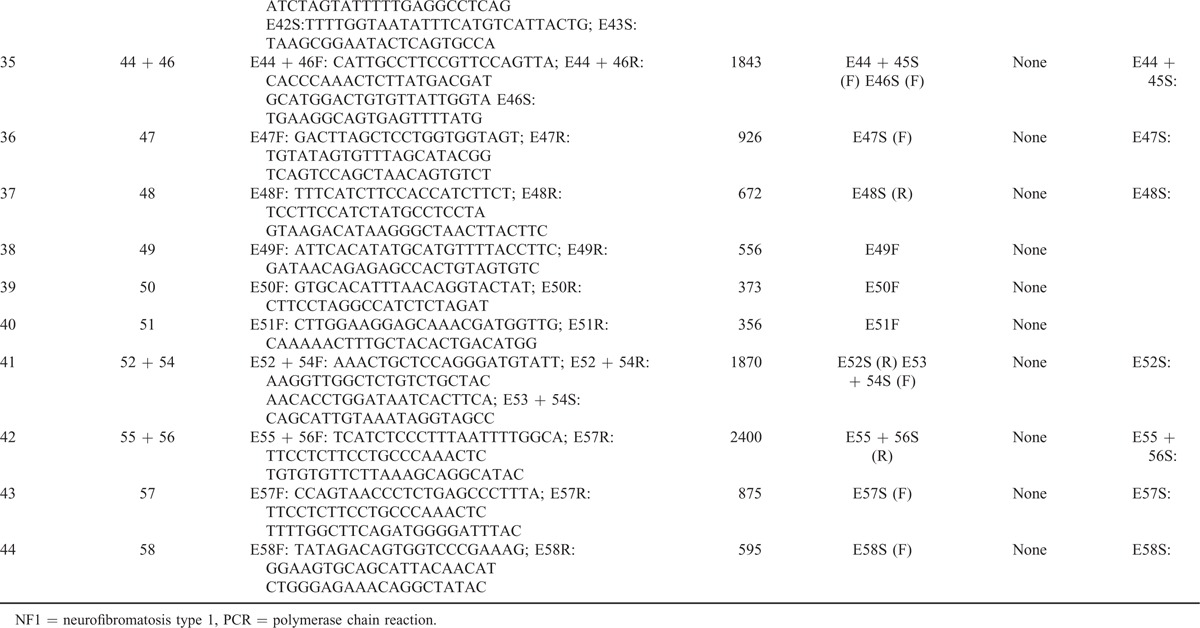
DNA Primers

### *NF1* Mutation Analysis by a DNA and cDNA Sequencing Approach

Using genomic DNA samples, all coding exons and intron–exon boundaries of the *NF1* gene were amplified by PCR with specific primers (Table 1  ) that were designed to avoid pseudogenes. PCR products were purified using a QIAquick PCR Purification Kit (Qiagen, Hilden, Germany) and were sequenced using an ABI PRISM3730 automated sequencer (Applied Biosystems, Waltham, MA). Mutations were identified by comparing with the corresponding genomic DNA reference sequence NC_000017.10. In addition, samples from unaffected parents and 120 unrelated population-match controls were sequenced for the detected mutation to exclude the possibility of polymorphisms in the *NF1* gene. cDNA sequencing was performed using extracted RNA samples to validate splice-site mutations, missense mutations, and mutations in large introns identified by DNA sequencing. The Expand Long Template PCR System (Roche, Basel, Switzerland) was used to amplify the full-length cDNA. A sequencing analysis was then performed for the same internal primers used for the above DNA sequencing. Mutation was identified by comparing with the corresponding cDNA reference sequence NM_000267.3. The primer oligonucleotide sequences for cDNA sequencing are provided in Table 2. The annealing temperature for long-range PCR primer sets was 51 to 64°C. A total of 20 ng of genomic DNA was amplified in a reaction volume of 20 μL containing 0.2 U *Taq*, 2 mM MgCl_2_, 0.5 μM forward and reverse primers, and 200 μM dNTPs. Amplification conditions were as follows: 94°C for 30 s, annealing temperature for 30 s, and 72°C for 1 min for 35 cycles. The extension for *NF1*-E36 was 30 s and for *NF1*-E55 + 56 was 2 min.

**TABLE 2:**
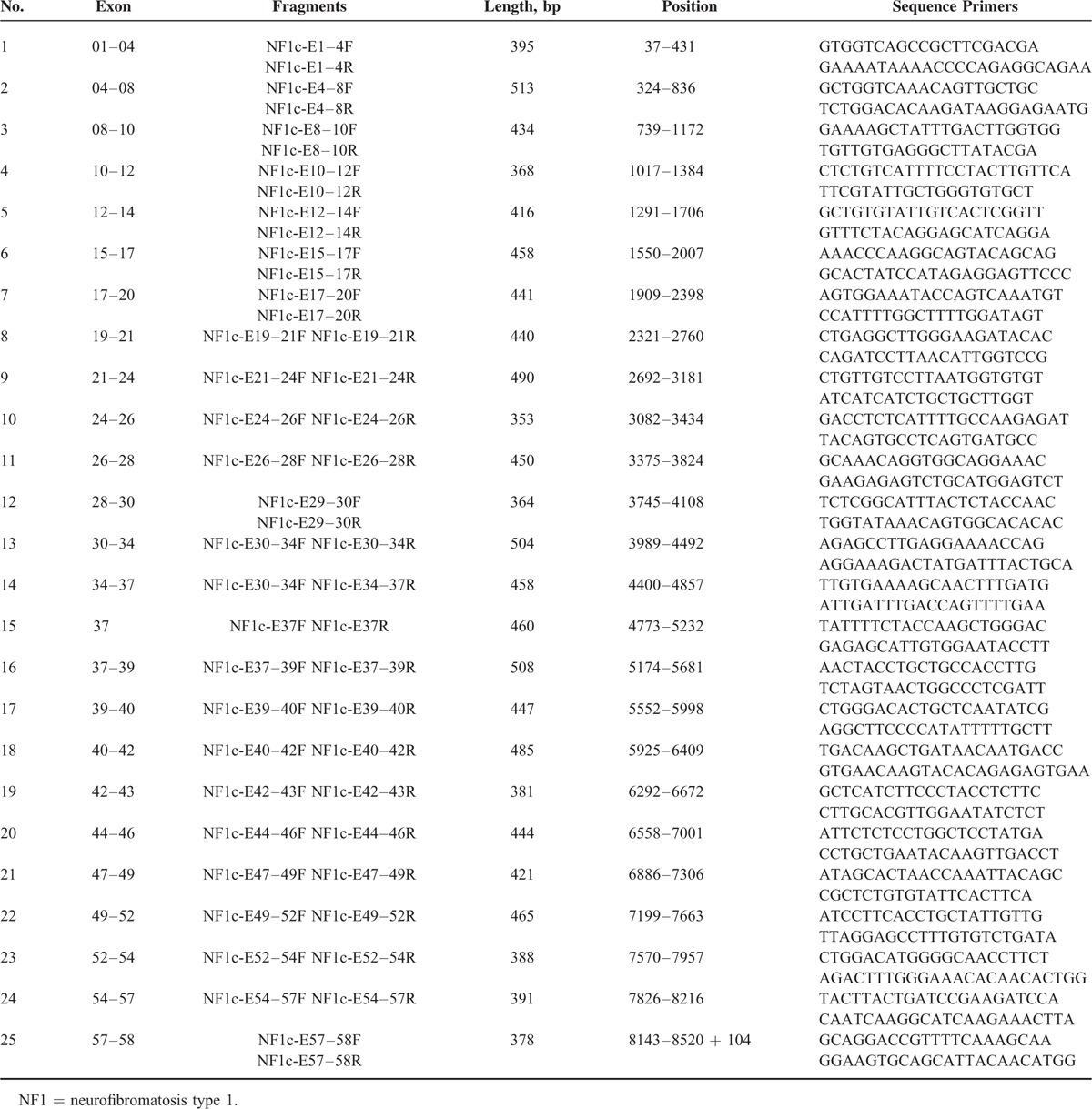
cDNA Primers

### Multiplex Ligation-Dependent Probe Amplification Analysis

When no pathogenic mutations were detected by both DNA and cDNA sequencing, the samples were analyzed using multiplex ligation-dependent probe amplification (MLPA) with the SALSA MLPA P081/P082 *NF1* Kits to detect single and multiple exon deletions/duplications and the SALSA MLPA P122 *NF1* AREA Kit to screen gross deletions in the *NF1* chromosomal region, according to the manufacturer's instructions (MRC Holland, Amsterdam, The Netherlands). Denatured genomic DNA (100 ng/150 ng) was added to the MLPA mix and the probes were allowed to anneal overnight before the subsequent ligation reaction was performed. qPCR amplification was performed using 6-carboxyfluoescein (FAM)-labeled primers; products were sequenced by an ABI Prism 3130 automatic DNA sequencer (Life Technologies, Saint Aubin, France). Peak areas for each separated fragment were measured by using Coffalyser.NET software (MRC Holland). Each MLPA product was normalized by dividing each peak area by the total peak area of reference probes peak for the sample to obtain the relative peak area values. The change of the peak values greater than ±0.3 was considered a duplication (an increase in value) or a deletion (a decrease in value). Ratios of <0.65 and >1.35 indicated deletions and duplications, respectively.

### Statistical Analyses

In silico prediction of the identified variants was performed using online prediction tools. Polymorphism Phenotyping v2 (PolyPhen-2: http://genetics.bwh.harvard.edu/pph2) was used to analyze missense changes.^20^ PolyPhen scores were interpreted as follows: benign, 0.00 to 0.20; possibly damaging, 0.20 to 0.85; and probably damaging, 0.85 to 1.00.

## RESULTS

**FIGURE 1:**
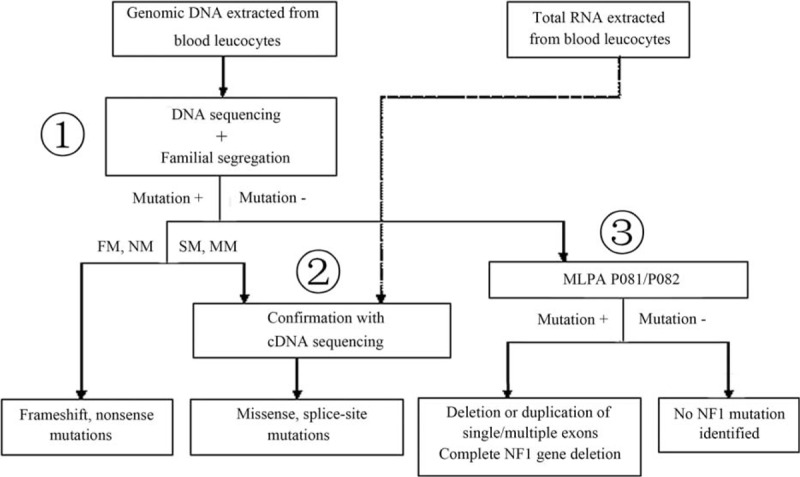
Flow chart for comprehensive *NF1* mutation detection. Point mutations identified by DNA sequencing with specific primers (step 1) represented 68.8% of the *NF1* mutations. Frameshift and nonsense mutations were identified in 31.3% and 18.8% of NF1 patients, respectively. In addition, missense and splice-site mutations were confirmed using cDNA sequencing (step 2) and were observed in 12.5% and 6.3% of NF1 patients, respectively. In the case of a negative result using DNA sequencing, an analysis of *NF1* complete and large partial deletions was performed using multiplex ligation-dependent probe amplification (MLPA) (step 3) and occurred in 25.0% of NF1 patients. This comprehensive mutation screening procedure enabled us to identify an *NF1* mutation in 93.8% of the NF1 patients in our study. NF1 = neurofibromatosis type 1.

A comprehensive protocol was used to screen mutations for 32 NF1 patients by sequencing at the both DNA and cDNA levels using MLPA and familial segregation analyses (Figure 1). Our method failed to detect any classic *NF1* mutations in 2 index patients with clear NF1 phenotypes based on the National Institutes of Health (NIH) diagnostic criteria.

### Clinical Manifestations in NF1 Patients

**FIGURE 2:**
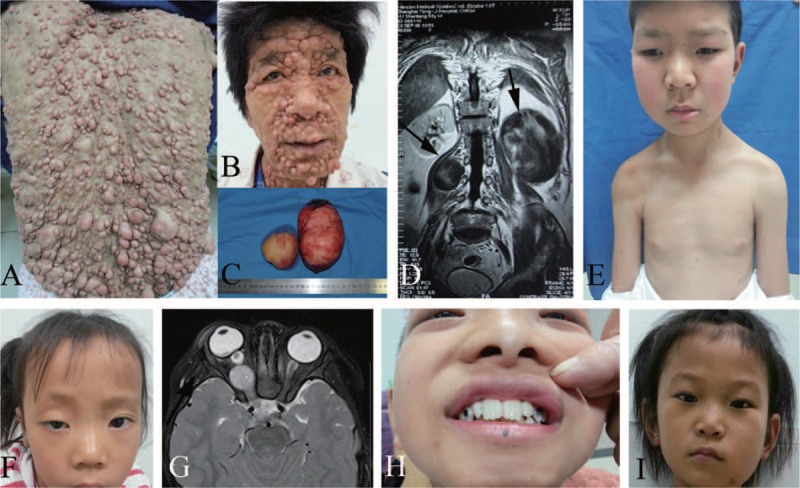
Clinical manifestations of the patients. Gross generalized cutaneous neurofibromas (CN) on the back (A) and face (B) of patient 28. (C) Two malignant peripheral nerve sheath tumors (arrows) from patient 18 with 7-cm and 9.5-cm diameters. (D) Trunk axial MRI shows 2 low-signal-intensity lesions in T1-weight imaging. (E) Macrocephaly in patient 27. (F) Optic gliomas (OG) in patient 17. (G) Orbital axial CT of patient 17 shows OG of her right eye (arrow). (H) Dental caries in patient 26. (I) Facial dysmorphism in patient 2. CT = computed tomography, MRI = magnetic resonance imaging.

The observed clinical manifestations (Figure 2) of 32 patients with NF1 are summarized in Table 3. CALs and skin-fold freckling were observed in most of the patients (96.9% and 90.6%, respectively). Subcutaneous neurofibromas, cutaneous neurofibromas, and Lisch nodules were observed in 18 (56.3%), 17 (50.0%), and 19 (59.4%) patients, respectively. Patient 28 had gross generalized cutaneous neurofibromas all over her body. Plexiform neurofibromas were observed in 14 (43.8%) patients. Malignant peripheral nerve sheath tumors were both identified in 2 (6.3%) patients. Five (15.6%) patients had mental retardation and 3 (9.4%) patients suffered from optic gliomas. Four rare symptoms (scoliosis, macrocephaly, dental caries, and facial dysmorphism) were all identified in 1 (3.1%) patient.

**TABLE 3:**
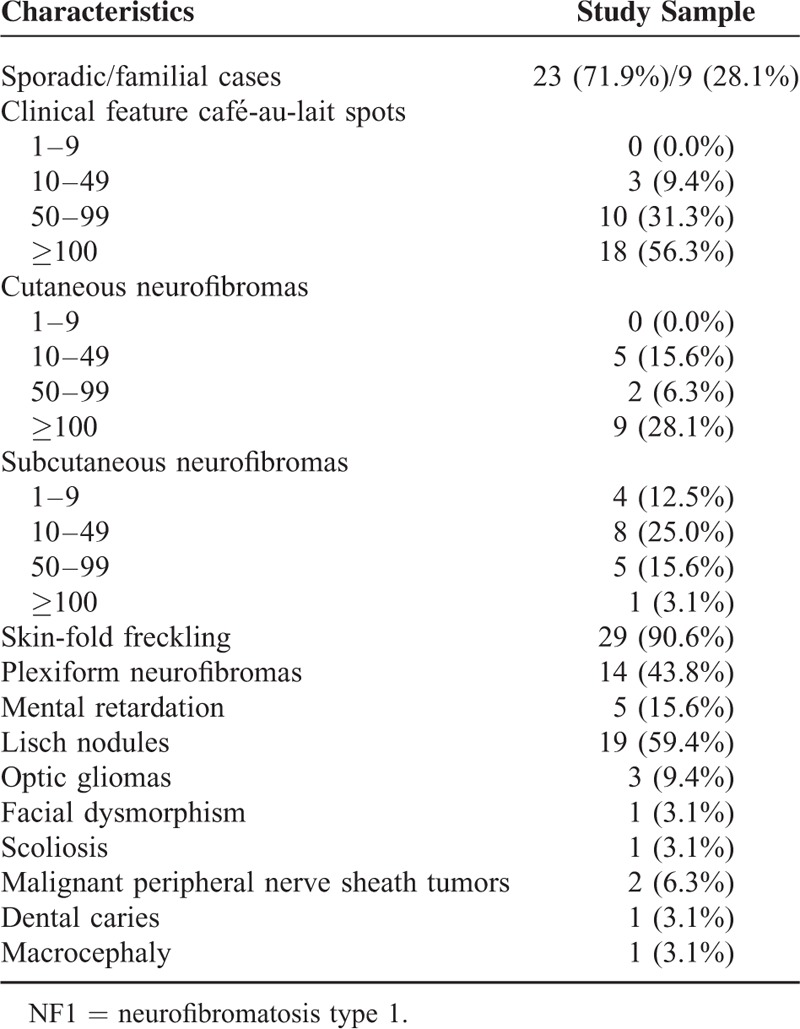
Clinical Characteristics of 32 NF1 Patients

### *NF1* Mutation Spectrum

*NF1* mutations were identified in 30 of the 32 patients (93.8%). The mutation analysis showed a wide spectrum of *NF1* mutations in the cohort (Table 4). The identified mutations were evenly distributed across exons 4 through 54 and intron 32 of the *NF1* gene. The spectrum of mutations included 8 large deletions, which were detected in the MLPA analysis. Frameshift mutations were found in 10 (33.3%) patients and nonsense mutations were identified in 6 (20.0%) patients. Direct DNA sequencing revealed 4 (13.3%) missense mutations and 2 (6.7%) splicing mutations. Using cDNA sequencing, all of the above point mutations were confirmed except for c.3113+1G>A, and none of the missense mutations resulted in a new splice site. All of the missense changes were predicted to be “probably damaging” with a PolyPhen score of 0.972 to 0.991 (benign: 0.00–0.20, possibly damaging: 0.20–0.85, probably damaging: 0.85–1.00).

**TABLE 4:**
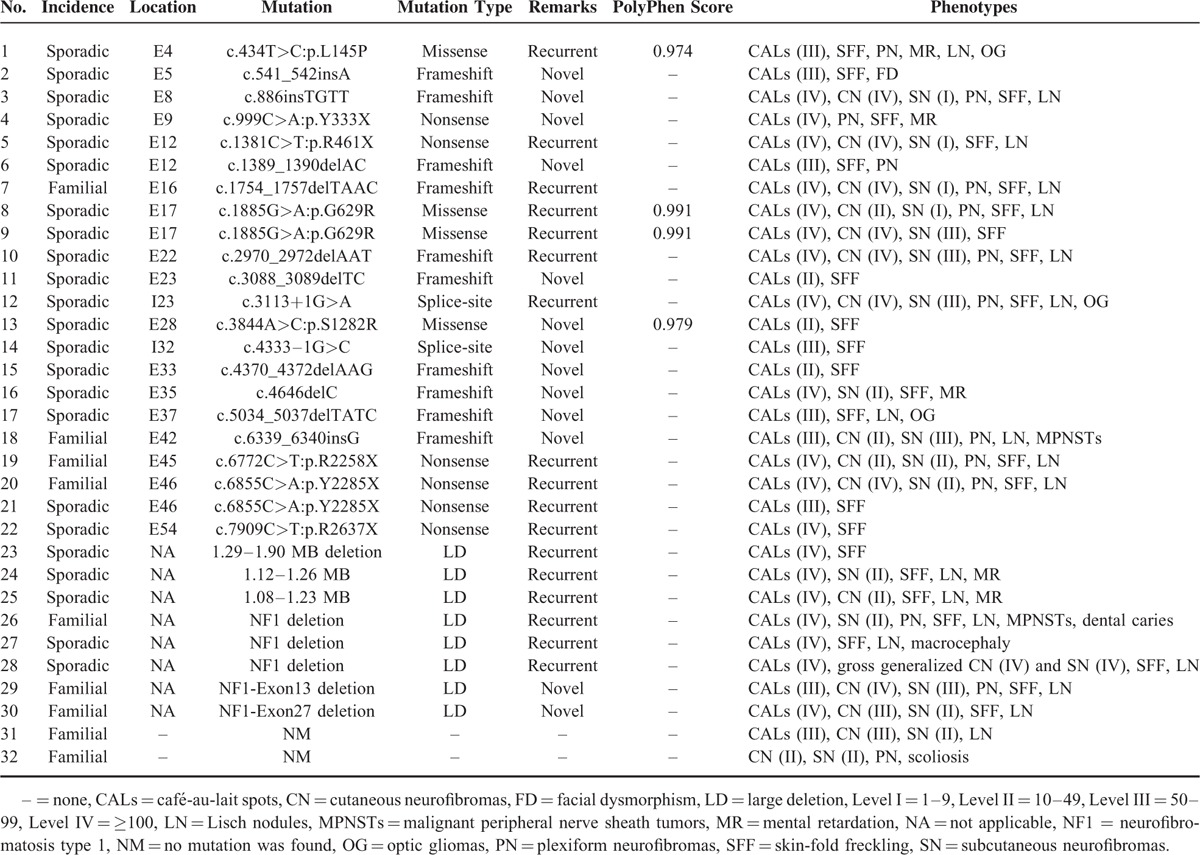
Mutations Identified in NF1 and Clinical Features of the Patients in This Study

Thirteen (43.3%) mutations were novel. Each of these is listed in Table 4. Among all mutations, only 2, c.1885G>A (p.G629R) and c.6855C>A (p.Y2285X), were recurrent (i.e., they were identified in 2 unrelated patients). This indicated that there was no hot-spot for mutations in the *NF1* gene.

### Genotype–Phenotype Correlations

Genotype–phenotype correlations were evaluated in 30 unrelated NF1 patients with 8 large deletions, 10 frameshift mutations, 6 nonsense mutations, 4 missense mutations, and 2 splicing mutations. Nine patients were familial cases and the others were sporadic cases. In total, 43.3% (13/30) of patients had de novo mutations. This result is consistent with previous estimates (∼50%) reported in the literature. However, the results do not indicate that phenotypes of sporadic cases were more severe than those of familial cases. In our study, patients with large deletions had severe clinical phenotypes. Based on a genotype–phenotype correlation analysis, we found that patients with identical mutations (patients 20 and 21 and patients 8 and 9) had wide phenotypic variation. Additionally, patients with identical symptoms, such as plexiform neurofibromas, did not have similar mutations with respect to type or location. These results do not indicate a clear relationship between specific *NF1* mutations and clinical phenotypes in our study.

## DISCUSSION

Despite the development of several methods for screening *NF1* mutations, it has been difficult to determine the genetic basis and genotype–phenotype associations for NF1. We established a novel protocol for the molecular diagnosis of NF1 that combines specific primers for PCR, sequencing at the both cDNA and DNA levels, MLPA, and familial segregation analyses. Our aim was to determine a sensitive, feasible, and comparatively economical protocol to detect *NF1* mutations from blood samples.

The identification of *NF1* mutations requires PCR amplification and analyses of all exons. However, highly homologous unprocessed pseudogenes hinder the amplification of exons. Screening at the cDNA level is a simple method to address this issue. Additionally, cDNA sequencing requires fewer samples than DNA sequencing. It can be used to rule out missense mutations at endonuclease sites that are splice-site mutations. However, cDNA sequencing has some disadvantages. First, it is difficult to design a cDNA-PCR amplicon that includes the complete region of *NF1* owing to the high CG content of the 5′ UTR region and its particular melting profile. Accordingly, we cannot design specific primers for the first exon for PCR amplification. Second, some splice-site mutations are not found at canonically conserved splice sites and may be located in introns.^15,21^ Such mutations cannot be detected by direct sequencing at the cDNA level. Third, nonsense-mediated mRNA decay (NMD) is a surveillance mechanism by which cells recognize and degrade mRNAs containing premature translation termination codons. Accordingly, some mutations might be missed owing to NMD; the mutation c.3113+1G>A in our study was not detected using cDNA sequencing, and this might be related to NMD. Finally, it is more difficult to preserve RNA samples than DNA samples, and the procedures for cDNA sequencing are cumbersome.

*NF1* is one of the largest genes in the human genome and has highly homologous unprocessed pseudogenes. This level of complexity makes it difficult to design appropriate primers for DNA sequencing. In addition, the number of samples needed for DNA sequencing is much larger than the number needed for cDNA sequencing. To address these issues, we designed highly specific primers based on the differences between the sequences of the pseudogenes and *NF1*; using these specific primers, we were able to successfully sequence the locus. Although the total number of PCRs needed for DNA sequencing is more than for cDNA sequencing, the total cost is not high owing to the lower price of DNA sequencing. So it is economical as compared to the previous price of DNA sequencing. Recently, Okumura et al^22^ have reported a practicable and inexpensive *NF1* mutation screening system based on CEL endonuclease-mediated heteroduplex incision with polyacrylamide gel electrophoresis and silver staining recently. Despite all that the present study could not adopt this technique because it was a new protocol that it had not been widely used in China. For these reasons, we used DNA sequencing with our specific PCR primers combined with MLPA to perform a mutation analysis of the *NF1* gene, and cDNA sequencing was used as a complement to verify splice-site mutations, missense mutations, and mutations located in large introns identified by DNA sequencing. Using this protocol, we identified mutations in 30 out of 32 (93.8%) NF1 patients who met the NIH criteria for diagnosis, including 10 (33.3%) frameshift, 4 (13.3%) missense, 6 (20.0%) nonsense, and 2 (6.7%) splice site mutations. Eight (26.7%) gross deletions involving more than 1 exon were also identified. In summary, we designed a highly specific, feasible, comparatively economical protocol for the routine molecular diagnosis of NF1, achieving 93.8% sensitivity (Figure 1).

For patients with intragenic *NF1* mutations (more than 90% of all NF1 cases), no genotype–phenotype correlations have been established to date,^11–14^ except that NF1 patients with an *NF1* microdeletion have more severe clinical phenotypes, including a higher prevalence of learning disabilities and dysmorphic features.^8,9,23^ This is consistent with the results of our study, in which patients with large deletions had severe clinical phenotypes. In particular, gross generalized cutaneous neurofibromas were observed in patient 29, and these were more severe than those of other patients with large deletions.

In conclusion, we designed a sensitive, feasible, and comparatively economical protocol to detect *NF1* mutations; when it was applied to patients who fulfilled the NIH diagnostic criteria for NF1, we observed mutations at a rate of 93.8%. Using our protocol, 30 distinct *NF1* mutations were identified in 32 patients, and 13 were novel.
